# Multimodal management and complete resection of invasive Type B3 thymoma with vascular reconstruction: a case report

**DOI:** 10.1186/s13019-024-03318-1

**Published:** 2025-01-07

**Authors:** Puiyee Sophia Chan, Akshay J. Patel, Gianluca Lucchese, Andrea Bille

**Affiliations:** 1 Department of Thoracic Surgery, Guy’s and St Thomas’ NHS Foundation Trust, London, England; 2 Institute of Immunology and Immunotherapy, University of Birmingham, Vincent Drive, Edgbaston, B15 2TT England; 3 Department of Cardiac Surgery, Guy’s and St Thomas’ NHS Foundation Trust, London, England

**Keywords:** Thymic epithelial tumours (TET), Thymoma, Chemoradiation, Neoadjuvant, Redo surgery

## Abstract

**Introduction:**

Thymomas and thymic carcinomas are rare anterior mediastinal tumours, accounting for 0.2–1.5% of all cancers. Surgical resection is key to treatment, though invasion of surrounding structures like great vessels can complicate this. This case report details the management of a type B3 thymoma (T4 N0 M0) in a 41-year-old male.

**Case report:**

A 41-year-old male presented with myasthenic symptoms and was diagnosed with a large thymic mass involving the brachiocephalic vein and superior vena cava. After 4 cycles of neoadjuvant chemotherapy, partial resection was performed, followed by radiotherapy. Residual disease led to a second surgery, during which tumour resection and vascular reconstruction using cardiopulmonary bypass were successfully completed. Postoperative recovery was uneventful.

**Discussion:**

Complete resection, including re-resection, when necessary, is crucial for improved outcomes in thymoma patients. Even with great vessel invasion, aggressive surgery, coupled with chemotherapy and vascular reconstruction, can achieve good survival outcomes.

**Conclusion:**

Multimodal management, including chemotherapy, complete resection, and vascular reconstruction, offers the best prognosis for invasive thymomas, even with great vessel involvement.

## Introduction

Thymomas and thymic carcinomas (thymic epithelial tumours (TETs)) are the most common tumours of the anterior mediastinum. However, they are relatively rare malignancies that account for only about 0.2–1.5% of all cancers [1]. Surgical resection forms the mainstay of treatment for these lesions and in the advanced setting, it is a core component of multi-modality treatment [2]. However, locally invasive thymomas can spread to surrounding organs and structures such as the great vessels, making surgical resection challenging. Increased rates of incomplete resection results in increased rates of recurrence, making it essential to adopt a multimodality therapeutic approach in order to achieve the best chance of complete resection and prolong the survival of patients with invasive thymomas. We present a case of a patient with an incidental diagnosis of type B3 Thymoma T4 N0 M0 after initially presenting to hospital in May 2023 with a viral infection. The tumour had already invaded surrounding vasculature including the brachiocephalic vein and superior vena cava on presentation. The patient then underwent 4 cycles of neoadjuvant cyclophosphamide, doxorubicin, cisplatin (CAP) chemotherapy followed by the first attempt at resection and radiotherapy before complete resection was achieved.

## Case report

A previously fit and well 41-year-old male patient presented to the Emergency Department (May 2023) with worsening symptoms of impaired speech, difficulty swallowing, difficulty breathing and fatigue in his limbs. In the preceding weeks he was suffering with a viral upper respiratory tract infection. He was subsequently treated for myasthenic crisis; over a 2-week period, he received IV immunoglobulin, prednisolone and pyridostigmine for his symptoms.

A CT chest showed an incidental finding of a large 12 cm x 6.3 cm x 11.4 cm thymic mass invading the lungs and right atrial appendage, partial encasement of the left brachiocephalic vein and compression of the superior vena cava and right superior pulmonary vein. A PET CT showed the mass as being highly FDG avid (SUVmax 9.8). These findings were consistent with a thymic malignancy (T4N0M0) and pre-operative histology showed a type B thymoma (AE1/AE3, p63 positive, TTF-1 negative). Blood tests showed results of acetylcholine receptor antibody > 20, antinuclear antibody positive, SS-A antibody 3.2 (high) and SS-A60 antibody 3.2 (high).

The patient underwent 4 cycles of CAP chemotherapy and prophylactic granulocyte colony stimulating factor was administered with each cycle (21 days between cycles). A mid-treatment scan (post 3 cycles) showed marginal reduction in the size of the mediastinal mass and the patient was deemed suitable for surgery. The patient underwent an exploratory median sternotomy and debulking of the primary mass. The tumour was unable to be fully excised as it was found to have enveloped the superior vena cava and innominate vein, and it was challenging to gain control of these vessels for resection (R2 resection). The multidisciplinary team (MDT) thence commenced him on adjuvant radiotherapy (60 Gy in 30 fractions). During this period, he suffered with worsening myasthenic symptoms and developed steroid-induced Cushing’s syndrome.

**Fig. 1:**
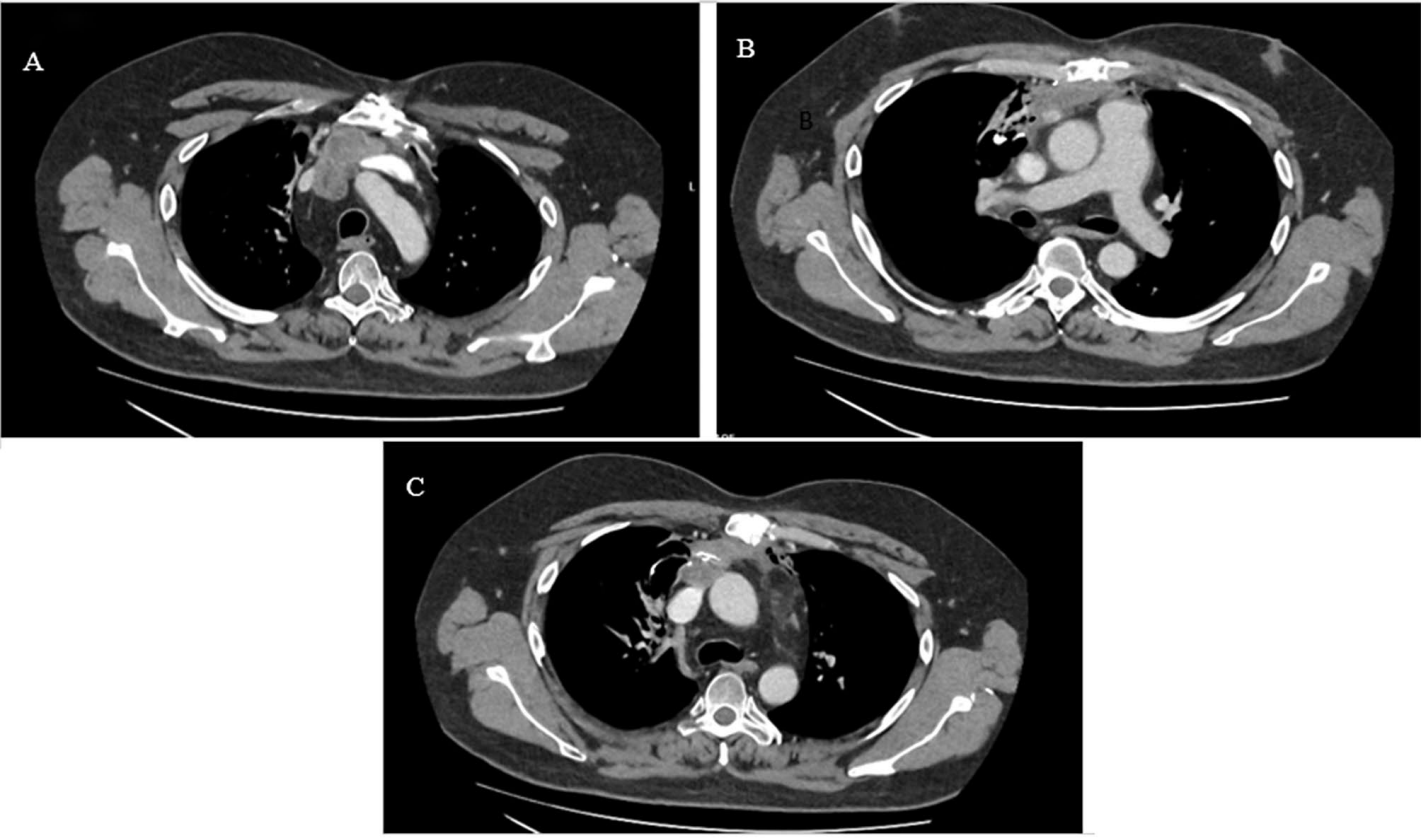
Contrast-enhanced CT axial section of anterior mediastinal mass post initial chemotherapy and 1st debulking operation **(A)**. Mass at the level of the PA bifurcation and Right Ventricular outflow tract (RVOT) **(B)**. Mass at the level of the carina **(C)**

**Fig. 2:**
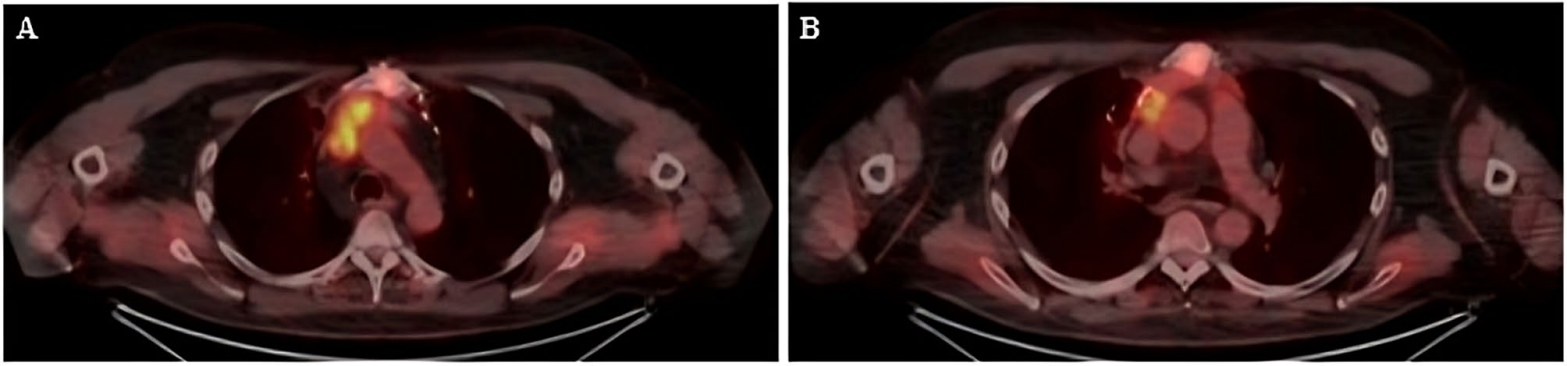
CT-PET axial sections showing avidity of the mass throughout the mediastinum **(A)**. Avidity of the tail of the mass below the subcarina **(B)**

**Fig. 3:**
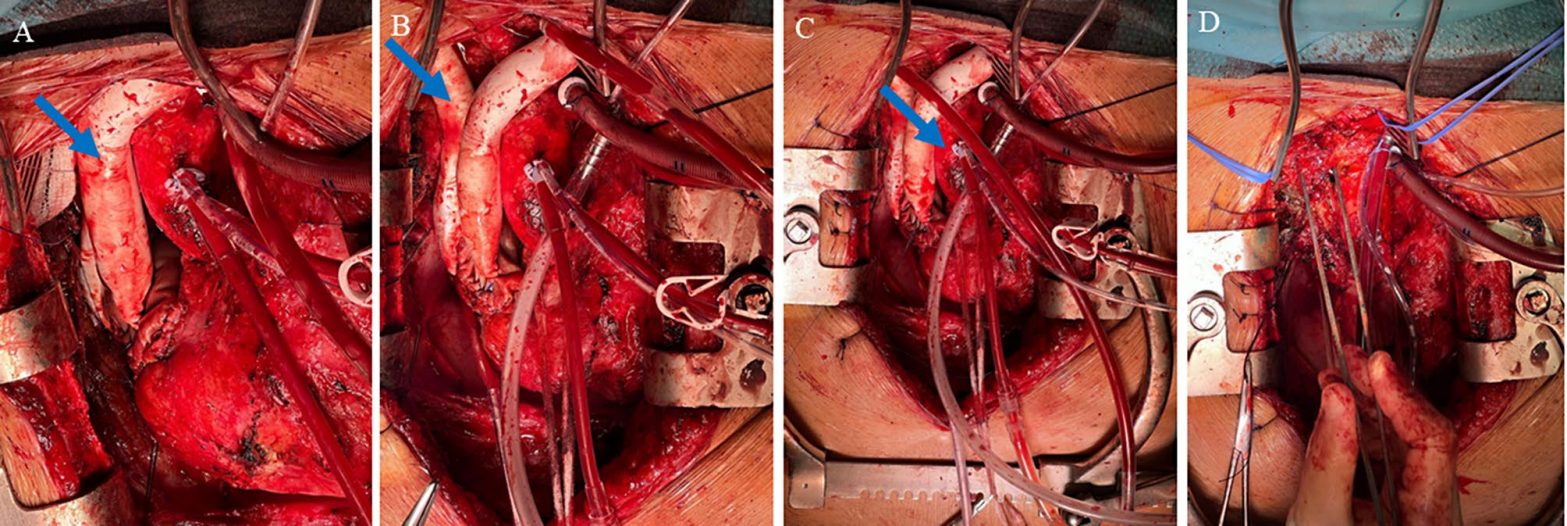
Intra-operative images showing the reconstruction of the major venous vasculature (**A** and **B**). Blue arrow in panel A indicates the innominate vein graft. Blue arrow in panel B indicates the SVC graft. Blue arrow in panel C indicates the ascending aorta and site of cannulation. Cannulation of the ascending aorta for reconstruction of the right atrium **(C)**. Peri-aortic tissue and superior mediastinal mass being resected **(D)**

Follow-up imaging (Figs. 1 and 2) demonstrated evidence of residual local disease and patient was keen to proceed with redo sternotomy with tumour resection and vascular reconstruction. At this point the patient was referred to our centre and underwent a redo operation with residual thymoma resection, reconstruction of the right atrium, superior vena cava, and extra-anatomical bypass from the innominate vein directly to the right atrial appendage (Fig. 3). Peri-aortic tissue was dissected and sent for frozen section which tested positive for tumour, following which further dissection was completed. Due to extensive invasion of the tumour into the right atrium, a decision was made for the patient to be put on cardiopulmonary bypass in order to resect further tissue isolate and clamp the superior vena cava at its origin. The heart was arrested using antegrade cardioplegia to mitigate the risk of paradoxical air embolism, given the potential presence of a patent foramen ovale on the transoesophageal echocardiography that had been done prior to the surgery. The right atrium was opened and inspected however no obvious patent foramen ovale was found. The tumour was excised *en bloc*, including the superior vena cava, a portion of the right atrium and the innominate vein. A wedge resection of the lung was performed and margins from the lung, atrium, distal superior vena cava and innominate vein all returned negative on frozen section. The superior vena cava was reconstructed with 16 mm Gore-Tex conduit with an end-to-end anastomosis to the right subclavian-right jugular confluence proximally, and an end-to-end anastomosis to the right atrium distally using a continuous Prolene 4 − 0 running suture with side clamping. The innominate vein was connected to the right atrium using a 16 mm extra-anatomical Gore-Tex conduit. This involved an end-to-end anastomosis with the innominate vein proximally and the right atrium distally on a reinforced glutaraldehyde-fixed bovine pericardial patch. The anastomoses were secured with a continuous Prolene 4/0 running suture. Cardiopulmonary bypass was successfully weaned with the heart in normal sinus rhythm and minimal inotropic support. A left ventricular pacing wire was positioned and secured, with routine sternal closure. The patient was admitted to the intensive care unit for close monitoring. Drains were removed on day 4 postoperatively. The myasthenic symptoms remained under control with an increased steroid regimen and regular input from neurology.

## Discussion

Complete surgical resection of thymomas is widely accepted to be one of the factors that is most associated with better outcomes and increased length of survival of the patient [3, 10]. Surgical resection should therefore be attempted in all resectable thymomas, with the use of neoadjuvant chemotherapy in order to achieve up front biological control and better chance of R0 resection in advanced stage tumours [4]. The “completeness” of the resection has been shown to be one of the key factors associated with prolonged survival, including the necessity of complete re-resection of recurrent thymomas for the best prognosis [5].

However, even with complete resections, 10–30% of patients develop a recurrence and require further surgical resection or medical treatment. A quantitative meta-analysis _(6)_ assessing the outcomes in patients with recurrent thymomas who then underwent surgical re-resection, showed a significant increase in 5-year overall survival rates compared to patients who had been treated with other therapies. Furthermore, a separate study showed that the outcomes in patients who had a complete resection for their thymoma recurrence versus patients who had only had incomplete resections were significantly better, with a 5-year survival rate of 90.9% compared to 44.7% [5]. Another study of 81 patients who underwent surgical resection for their recurrent thymomas, compared the 5- and 10-year survival rates between patients who had undergone complete and incomplete resections: 82.4% versus 55.9% and 65.4% versus 46.5% respectively [7]. This would suggest that redo surgeries and more radical complete resection should be attempted whenever possible to augment the odds of longer disease-free and overall survival [6, 8].

Historically tumours invading the great vessels were considered unresectable due to the high surgical risk and poor survival outcomes. In the modern era, owing to the availability of better prosthetic grafts, multi-disciplinary working and perioperative monitoring has improved outcomes in complex resections [9]. In a study comparing 54 patients with locally advanced thymoma, 20 of whom were given neo-adjuvant chemotherapy (NACT), this was found to be a favourable prognostic factor in overall patient survival [10] as it facilitated more complete surgical clearance of tumours that may have been deemed unresectable upfront [11]. A retrospective study comparing 20 patients with invasive thymoma invading the great vessels who underwent complete resection including vessel reconstruction, showed 3- and 5-year survival rates of 79.6% and 59.1% [12]. This was compared to a study showing the 5- and 10-year survival rates of patients with stage III thymoma who had only received other therapies such as radiotherapy, which were 53% and 44% [13]. This would suggest that complete resection is still the preferred therapy among patients with advanced thymomas invading surrounding great vessels, as a more aggressive surgical approach involving vascular reconstruction is both safe and results in significantly better survival outcomes.

Cardiopulmonary bypass was used in order to allow further resection of the tumour around the right atrium and great vessels. Studies have shown that this is safe, with no significant increase in risk of developing post-operative distant metastasis compared with patients who did not undergo cardiopulmonary bypass [9, 14, 15]. Our patient was started on warfarin and low molecular weight heparin to bridge post operatively. A retrospective analysis of 16 patients who underwent prosthetic grafts and were discharged on 3–6 months of warfarin, observed that all patients who had interrupted their warfarin and hence been switched to aspirin or clopidogrel, had experienced graft occlusion. This would suggest that long term anticoagulation therapy is necessary to prevent graft thrombosis, although no optimal length of anticoagulation has been agreed [16]. We chose to administer warfarin for the patient in this case due to the high risk of thrombosis and relatively lower risk of bleeding.

## Conclusion

The management of thymomas must be well planned between a variety of specialties and multidisciplinary teams (MDTs) in order to achieve the best outcomes for the patient. The aim should be a multimodal therapeutic approach with early initiation of neoadjuvant therapies if this is necessary in order to allow the best attempt at complete surgical resection which ultimately is still the gold standard treatment which results in the best survival prognosis. More aggressive thymomas with invasion of the great vessels should not be seen as a contraindication for complete resection, and instead attempted at experienced centres with the use of cardiopulmonary bypass and vascular resection which has been shown to be acceptable and safe management options.

## Data Availability

No datasets were generated or analysed during the current study.
